# Impact of Engagement in Digitally Delivered Goal Setting on Dementia Risk in Participants with Mild Cognitive Impairment or Subjective Cognitive Decline

**DOI:** 10.1002/alz70860_100493

**Published:** 2025-12-23

**Authors:** Jessica G. Amos, Lidan Zheng, Sophie Andrews, Kaarin J. Anstey

**Affiliations:** ^1^ University of New South Wales, Sydney, NSW, Australia; ^2^ Neuroscience Research Australia (NeuRA), Sydney, NSW, Australia; ^3^ UNSW Ageing Futures Institute, Syndey, NSW, Australia; ^4^ Thompson Institute, University of the Sunshine Coast, Sunshine Coast, QLD, Australia; ^5^ ARC Centre for Excellence in Population Ageing Research, Sydney, NSW, Australia

## Abstract

**Background:**

Modifiable lifestyle factors offer potential to reduce dementia risk, underscoring the need to identify effective behaviour change techniques (BCTs), particularly as the format of interventions change to incorporate more digital solutions. Goal setting is a key BCT that may influence behaviour change for dementia risk reduction (DRR). This study examined whether engagement with goal setting in a digitally delivered intervention was associated with improved DRR behaviours in individuals with subjective cognitive decline (SCD) or mild cognitive impairment (MCI).

**Method:**

Participants with SCD or MCI who completed the intervention arm of the MyCOACH randomised controlled trial (Amos et al., 2023) were included (*n* = 135, female = 62.2%, M_age_ = 74.55). A novel framework was developed to evaluate the quality of self‐directed SMART goals from the e‐learning and engagement with practitioner‐led teleconference goal setting were also assessed. A hierarchical regression analysis examined whether engagement with goal setting predicted DRR behaviour change, together with the effects of psychological determinants (attitudes, norms, perceived behavioural control) and baseline dementia risk (low vs. high), while controlling for demographic variables (age, gender, education). Secondary analyses compared the impact of self‐directed versus practitioner‐led goal setting on specific behaviours. Behaviour change was measured as the difference in a modified Australian National University‐Alzheimer's Disease Risk Index score between baseline and immediate follow‐up.

**Result:**

Engagement in goal setting alone did not significantly predict behaviour change in this context. Higher baseline dementia risk was the only significant predictor of DRR improvements (β =‐.439, *p* < .001). Secondary analyses revealed that higher‐quality self‐directed SMART goals were associated with improved MIND diet adherence (β =.024, *p* =.038), but no associations were found for physical activity behaviours. Practitioner‐led goal setting showed no significant effects.

**Conclusion:**

Findings indicated that goal setting alone did not significantly predict overall behavioural changes for DRR. However, setting higher‐quality dietary goals may drive changes specific to MIND diet adherence. This underscores the need for future research to explore combining or refining BCTs to determine what works best for different behaviours relevant to DRR. Notably, the impact of goal setting on long‐term outcomes was not assessed here and remains to be explored.

